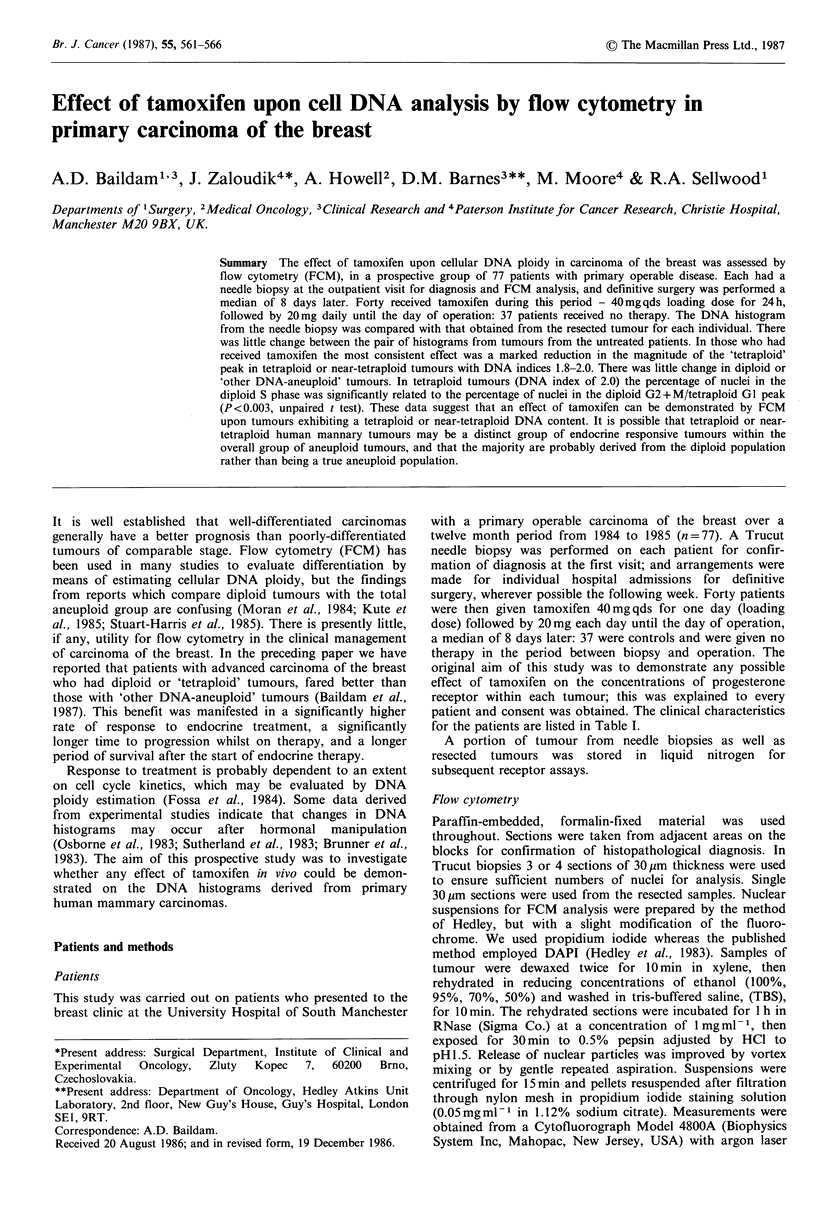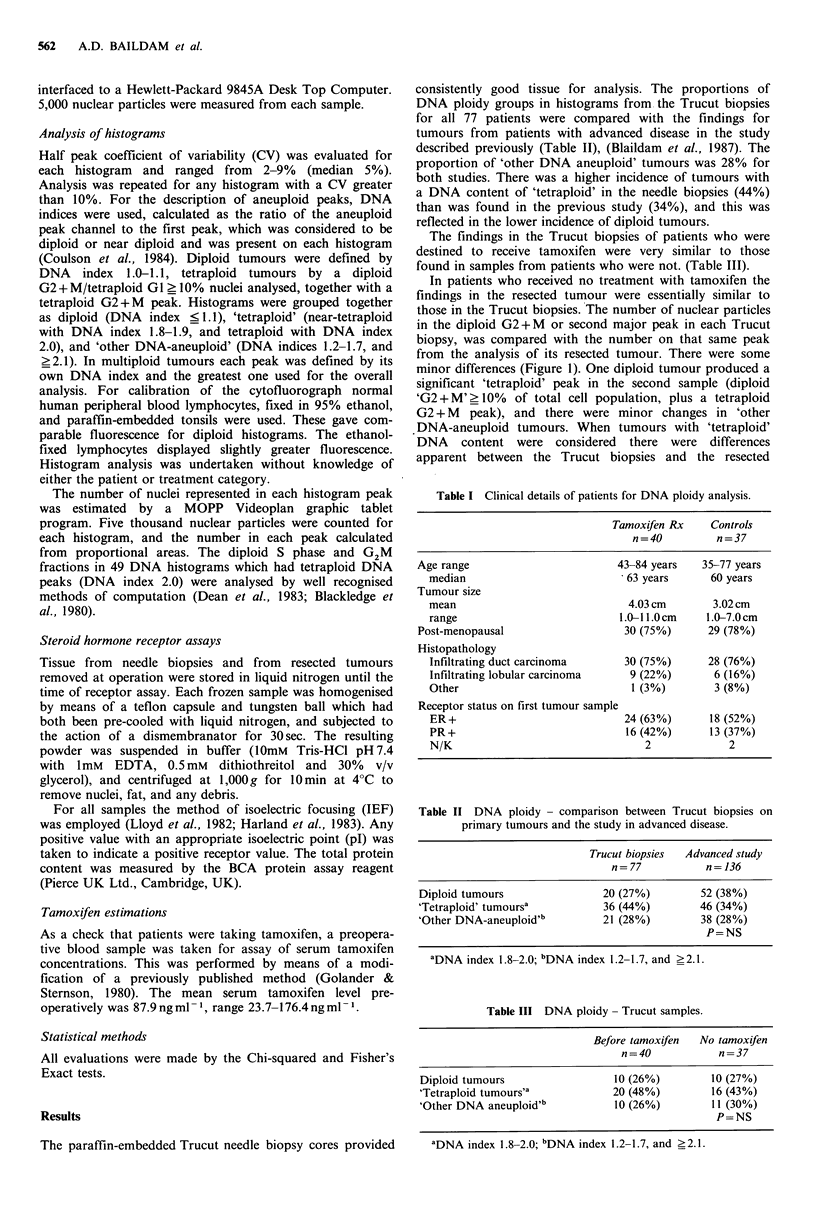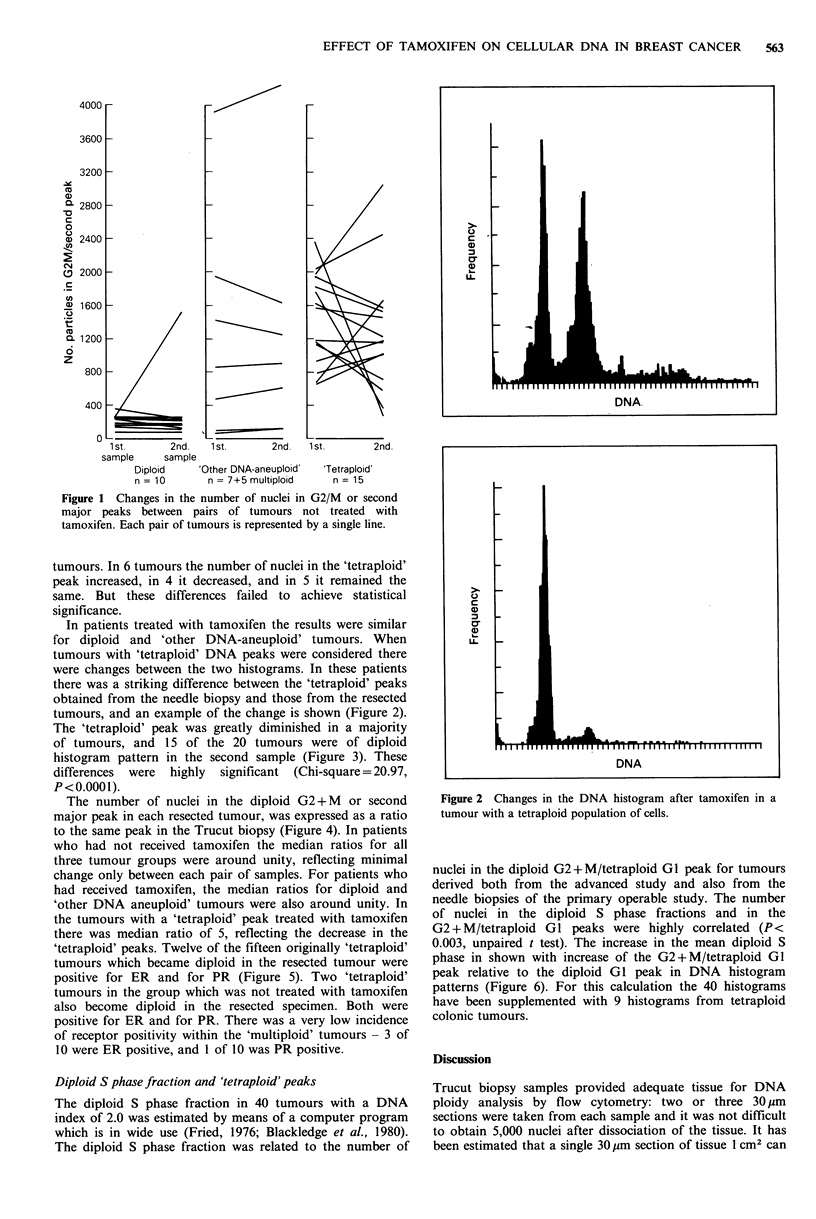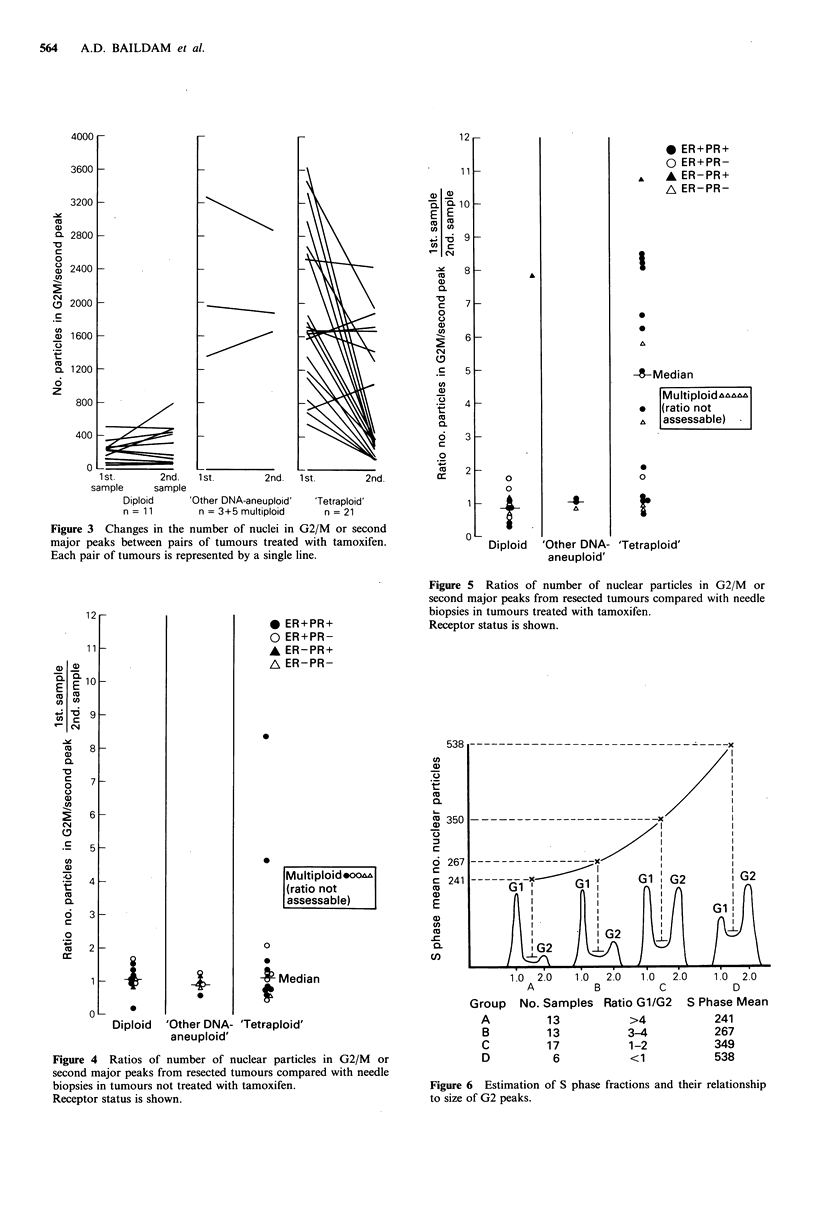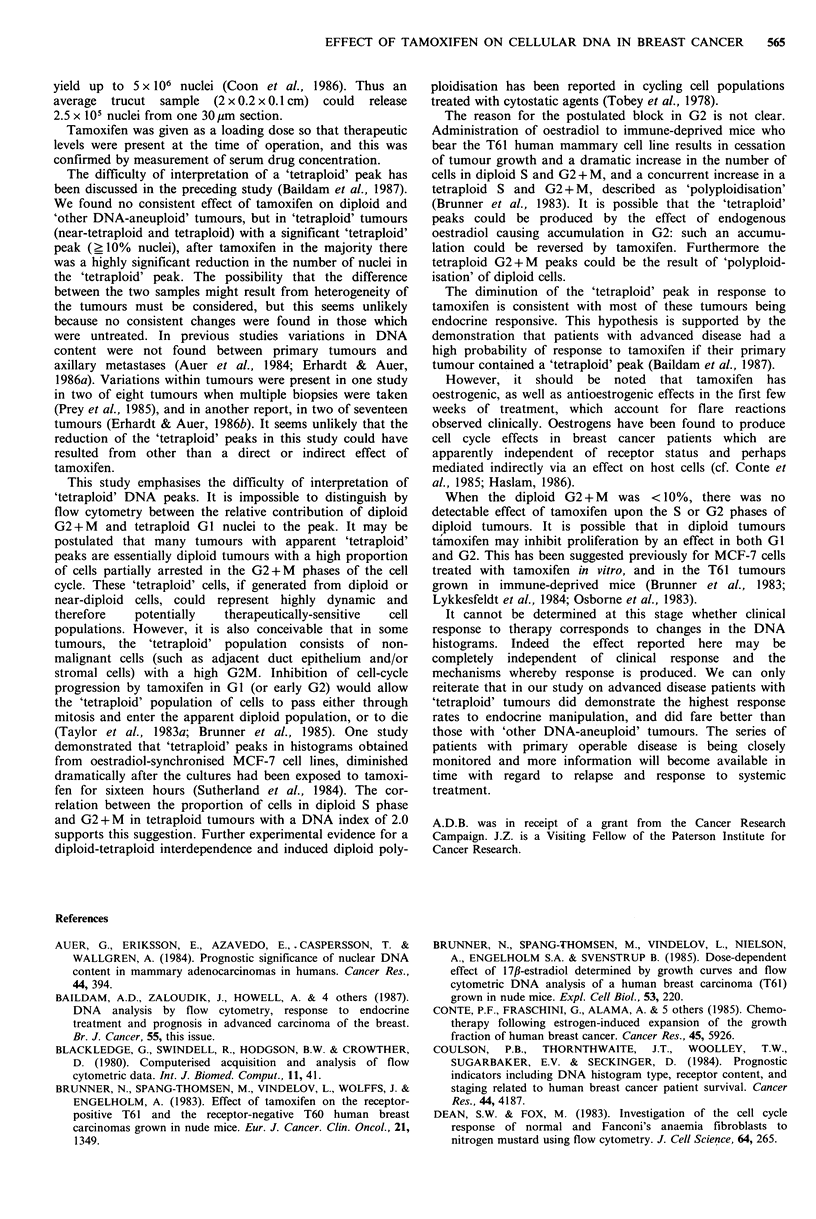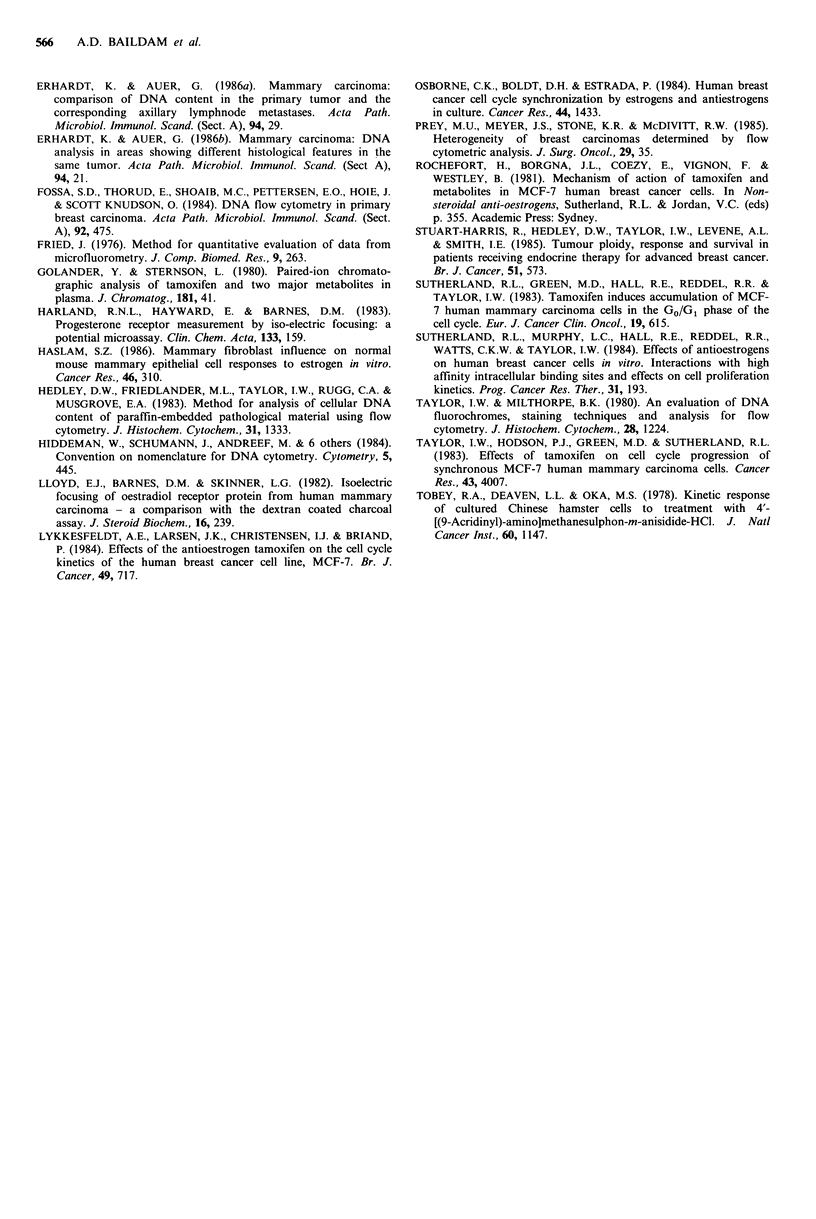# Effect of tamoxifen upon cell DNA analysis by flow cytometry in primary carcinoma of the breast.

**DOI:** 10.1038/bjc.1987.114

**Published:** 1987-05

**Authors:** A. D. Baildam, J. Zaloudik, A. Howell, D. M. Barnes, M. Moore, R. A. Sellwood

## Abstract

The effect of tamoxifen upon cellular DNA ploidy in carcinoma of the breast was assessed by flow cytometry (FCM), in a prospective group of 77 patients with primary operable disease. Each had a needle biopsy at the outpatient visit for diagnosis and FCM analysis, and definitive surgery was performed a median of 8 days later. Forty received tamoxifen during this period - 40 mg qds loading dose for 24 h, followed by 20 mg daily until the day of operation: 37 patients received no therapy. The DNA histogram from the needle biopsy was compared with that obtained from the resected tumour for each individual. There was little change between the pair of histograms from tumours from the untreated patients. In those who had received tamoxifen the most consistent effect was a marked reduction in the magnitude of the 'tetraploid' peak in tetraploid or near-tetraploid tumours with DNA indices 1.8-2.0. There was little change in diploid or 'other DNA-aneuploid' tumours. In tetraploid tumours (DNA index of 2.0) the percentage of nuclei in the diploid S phase was significantly related to the percentage of nuclei in the diploid G2 + M/tetraploid G1 peak (P less than 0.003, unpaired t test). These data suggest that an effect of tamoxifen can be demonstrated by FCM upon tumours exhibiting a tetraploid or near-tetraploid DNA content. It is possible that tetraploid or near-tetraploid human mammary tumours may be a distinct group of endocrine responsive tumours within the overall group of aneuploid tumours, and that the majority are probably derived from the diploid population rather than being a true aneuploid population.


*Br. J. Cancer* (1987), **55**, 561–566 © The Macmillan Press Ltd., 1987

### Effect of tamoxifen upon cell DNA analysis by flow cytometry in primary carcinoma of the breast

A.D. Baildam[1,3], J. Zaloudik[4*], A. Howell[2], D.M. Barnes[3**], M. Moore[4] & R.A. Sellwood[1]

*Departments of [1]Surgery, [2]Medical Oncology, [3]Clinical Research and [4]Paterson Institute for Cancer Research, Christie Hospital, Manchester M20 9BX, UK.*

**Summary** The effect of tamoxifen upon cellular DNA ploidy in carcinoma of the breast was assessed by flow cytometry (FCM), in a prospective group of 77 patients with primary operable disease. Each had a needle biopsy at the outpatient visit for diagnosis and FCM analysis, and definitive surgery was performed a median of 8 days later. Forty received tamoxifen during this period – 40 mg qds loading dose for 24 h, followed by 20 mg daily until the day of operation: 37 patients received no therapy. The DNA histogram from the needle biopsy was compared with that obtained from the resected tumour for each individual. There was little change between the pair of histograms from tumours from the untreated patients. In those who had received tamoxifen the most consistent effect was a marked reduction in the magnitude of the 'tetraploid' peak in tetraploid or near-tetraploid tumours with DNA indices 1.8–2.0. There was little change in diploid or 'other DNA-aneuploid' tumours. In tetraploid tumours (DNA index of 2.0) the percentage of nuclei in the diploid S phase was significantly related to the percentage of nuclei in the diploid G2+M/tetraploid G1 peak ($P<0.003$, unpaired $t$ test). These data suggest that an effect of tamoxifen can be demonstrated by FCM upon tumours exhibiting a tetraploid or near-tetraploid DNA content. It is possible that tetraploid or near-tetraploid human mannary tumours may be a distinct group of endocrine responsive tumours within the overall group of aneuploid tumours, and that the majority are probably derived from the diploid population rather than being a true aneuploid population.

It is well established that well-differentiated carcinomas generally have a better prognosis than poorly-differentiated tumours of comparable stage. Flow cytometry (FCM) has been used in many studies to evaluate differentiation by means of estimating cellular DNA ploidy, but the findings from reports which compare diploid tumours with the total aneuploid group are confusing (Moran *et al.*, 1984; Kute *et al.*, 1985; Stuart-Harris *et al.*, 1985). There is presently little, if any, utility for flow cytometry in the clinical management of carcinoma of the breast. In the preceding paper we have reported that patients with advanced carcinoma of the breast who had diploid or 'tetraploid' tumours, fared better than those with 'other DNA-aneuploid' tumours (Baildam *et al.*, 1987). This benefit was manifested in a significantly higher rate of response to endocrine treatment, a significantly longer time to progression whilst on therapy, and a longer period of survival after the start of endocrine therapy.

Response to treatment is probably dependent to an extent on cell cycle kinetics, which may be evaluated by DNA ploidy estimation (Fossa *et al.*, 1984). Some data derived from experimental studies indicate that changes in DNA histograms may occur after hormonal manipulation (Osborne *et al.*, 1983; Sutherland *et al.*, 1983; Brunner *et al.*, 1983). The aim of this prospective study was to investigate whether any effect of tamoxifen *in vivo* could be demonstrated on the DNA histograms derived from primary human mammary carcinomas.

### Patients and methods

### *Patients*

This study was carried out on patients who presented to the breast clinic at the University Hospital of South Manchester with a primary operable carcinoma of the breast over a twelve month period from 1984 to 1985 ($n=77$). A Trucut needle biopsy was performed on each patient for confirmation of diagnosis at the first visit; and arrangements were made for individual hospital admissions for definitive surgery, wherever possible the following week. Forty patients were then given tamoxifen 40 mg qds for one day (loading dose) followed by 20 mg each day until the day of operation, a median of 8 days later: 37 were controls and were given no therapy in the period between biopsy and operation. The original aim of this study was to demonstrate any possible effect of tamoxifen on the concentrations of progesterone receptor within each tumour; this was explained to every patient and consent was obtained. The clinical characteristics for the patients are listed in Table I.

A portion of tumour from needle biopsies as well as resected tumours was stored in liquid nitrogen for subsequent receptor assays.

### *Flow cytometry*

Paraffin-embedded, formalin-fixed material was used throughout. Sections were taken from adjacent areas on the blocks for confirmation of histopathological diagnosis. In Trucut biopsies 3 or 4 sections of 30 $\mu$m thickness were used to ensure sufficient numbers of nuclei for analysis. Single 30 $\mu$m sections were used from the resected samples. Nuclear suspensions for FCM analysis were prepared by the method of Hedley, but with a slight modification of the fluorochrome. We used propidium iodide whereas the published method employed DAPI (Hedley *et al.*, 1983). Samples of tumour were dewaxed twice for 10 min in xylene, then rehydrated in reducing concentrations of ethanol (100%, 95%, 70%, 50%) and washed in tris-buffered saline, (TBS), for 10 min. The rehydrated sections were incubated for 1 h in RNase (Sigma Co.) at a concentration of 1 mg ml$^{-1}$, then exposed for 30 min to 0.5% pepsin adjusted by HCl to pH1.5. Release of nuclear particles was improved by vortex mixing or by gentle repeated aspiration. Suspensions were centrifuged for 15 min and pellets resuspended after filtration through nylon mesh in propidium iodide staining solution (0.05 mg ml$^{-1}$ in 1.12% sodium citrate). Measurements were obtained from a Cytofluorograph Model 4800A (Biophysics System Inc, Mahopac, New Jersey, USA) with argon laser

*Present address: Surgical Department, Institute of Clinical and Experimental Oncology, Zluty Kopec 7, 60200 Brno, Czechoslovakia.
**Present address: Department of Oncology, Hedley Atkins Unit Laboratory, 2nd floor, New Guy's House, Guy's Hospital, London SE1, 9RT.
Correspondence: A.D. Baildam.
Received 20 August 1986; and in revised form, 19 December 1986.

interfaced to a Hewlett-Packard 9845A Desk Top Computer. 5,000 nuclear particles were measured from each sample.

*Analysis of histograms*

Half peak coefficient of variability (CV) was evaluated for each histogram and ranged from 2–9% (median 5%). Analysis was repeated for any histogram with a CV greater than 10%. For the description of aneuploid peaks, DNA indices were used, calculated as the ratio of the aneuploid peak channel to the first peak, which was considered to be diploid or near diploid and was present on each histogram (Coulson *et al.*, 1984). Diploid tumours were defined by DNA index 1.0–1.1, tetraploid tumours by a diploid G2+M/tetraploid G1 ≧ 10% nuclei analysed, together with a tetraploid G2+M peak. Histograms were grouped together as diploid (DNA index ≦1.1), 'tetraploid' (near-tetraploid with DNA index 1.8–1.9, and tetraploid with DNA index 2.0), and 'other DNA-aneuploid' (DNA indices 1.2–1.7, and ≧2.1). In multiploid tumours each peak was defined by its own DNA index and the greatest one used for the overall analysis. For calibration of the cytofluorograph normal human peripheral blood lymphocytes, fixed in 95% ethanol, and paraffin-embedded tonsils were used. These gave comparable fluorescence for diploid histograms. The ethanol-fixed lymphocytes displayed slightly greater fluorescence. Histogram analysis was undertaken without knowledge of either the patient or treatment category.

The number of nuclei represented in each histogram peak was estimated by a MOPP Videoplan graphic tablet program. Five thousand nuclear particles were counted for each histogram, and the number in each peak calculated from proportional areas. The diploid S phase and $G_2M$ fractions in 49 DNA histograms which had tetraploid DNA peaks (DNA index 2.0) were analysed by well recognised methods of computation (Dean *et al.*, 1983; Blackledge *et al.*, 1980).

*Steroid hormone receptor assays*

Tissue from needle biopsies and from resected tumours removed at operation were stored in liquid nitrogen until the time of receptor assay. Each frozen sample was homogenised by means of a teflon capsule and tungsten ball which had both been pre-cooled with liquid nitrogen, and subjected to the action of a dismembranator for 30 sec. The resulting powder was suspended in buffer (10mM Tris-HCl pH 7.4 with 1mM EDTA, 0.5 mM dithiothreitol and 30% v/v glycerol), and centrifuged at 1,000*g* for 10 min at 4°C to remove nuclei, fat, and any debris.

For all samples the method of isoelectric focusing (IEF) was employed (Lloyd *et al.*, 1982; Harland *et al.*, 1983). Any positive value with an appropriate isoelectric point (pI) was taken to indicate a positive receptor value. The total protein content was measured by the BCA protein assay reagent (Pierce UK Ltd., Cambridge, UK).

*Tamoxifen estimations*

As a check that patients were taking tamoxifen, a preoperative blood sample was taken for assay of serum tamoxifen concentrations. This was performed by means of a modification of a previously published method (Golander & Sternson, 1980). The mean serum tamoxifen level preoperatively was 87.9 ng ml$^{-1}$, range 23.7–176.4 ng ml$^{-1}$.

*Statistical methods*

All evaluations were made by the Chi-squared and Fisher's Exact tests.

### Results

The paraffin-embedded Trucut needle biopsy cores provided consistently good tissue for analysis. The proportions of DNA ploidy groups in histograms from the Trucut biopsies for all 77 patients were compared with the findings for tumours from patients with advanced disease in the study described previously (Table II), (Blaildam *et al.*, 1987). The proportion of 'other DNA aneuploid' tumours was 28% for both studies. There was a higher incidence of tumours with a DNA content of 'tetraploid' in the needle biopsies (44%) than was found in the previous study (34%), and this was reflected in the lower incidence of diploid tumours.

The findings in the Trucut biopsies of patients who were destined to receive tamoxifen were very similar to those found in samples from patients who were not. (Table III).

In patients who received no treatment with tamoxifen the findings in the resected tumour were essentially similar to those in the Trucut biopsies. The number of nuclear particles in the diploid G2+M or second major peak in each Trucut biopsy, was compared with the number on that same peak from the analysis of its resected tumour. There were some minor differences (Figure 1). One diploid tumour produced a significant 'tetraploid' peak in the second sample (diploid 'G2+M' ≧ 10% of total cell population, plus a tetraploid G2+M peak), and there were minor changes in 'other DNA-aneuploid tumours. When tumours with 'tetraploid' DNA content were considered there were differences apparent between the Trucut biopsies and the resected

**Table I** Clinical details of patients for DNA ploidy analysis.

| | *Tamoxifen Rx* *n=40* | *Controls* *n=37* |
|---|---|---|
| Age range | 43–84 years | 35–77 years |
| median | 63 years | 60 years |
| Tumour size | | |
| mean | 4.03 cm | 3.02 cm |
| range | 1.0–11.0 cm | 1.0–7.0 cm |
| Post-menopausal | 30 (75%) | 29 (78%) |
| Histopathology | | |
| Infiltrating duct carcinoma | 30 (75%) | 28 (76%) |
| Infiltrating lobular carcinoma | 9 (22%) | 6 (16%) |
| Other | 1 (3%) | 3 (8%) |
| Receptor status on first tumour sample | | |
| ER+ | 24 (63%) | 18 (52%) |
| PR+ | 16 (42%) | 13 (37%) |
| N/K | 2 | 2 |

**Table II** DNA ploidy – comparison between Trucut biopsies on primary tumours and the study in advanced disease.

| | *Trucut biopsies* *n=77* | *Advanced study* *n=136* |
|---|---|---|
| Diploid tumours | 20 (27%) | 52 (38%) |
| 'Tetraploid' tumours[a] | 36 (44%) | 46 (34%) |
| 'Other DNA-aneuploid'[b] | 21 (28%) | 38 (28%) |
| | | *P*=NS |

[a]DNA index 1.8–2.0; [b]DNA index 1.2–1.7, and ≧2.1.

**Table III** DNA ploidy – Trucut samples.

| | *Before tamoxifen* *n=40* | *No tamoxifen* *n=37* |
|---|---|---|
| Diploid tumours | 10 (26%) | 10 (27%) |
| 'Tetraploid tumours'[a] | 20 (48%) | 16 (43%) |
| 'Other DNA aneuploid'[b] | 10 (26%) | 11 (30%) |
| | | *P*=NS |

[a]DNA index 1.8–2.0; [b]DNA index 1.2–1.7, and ≧2.1.

**Figure 1** Changes in the number of nuclei in G2/M or second major peaks between pairs of tumours not treated with tamoxifen. Each pair of tumours is represented by a single line.

tumours. In 6 tumours the number of nuclei in the 'tetraploid' peak increased, in 4 it decreased, and in 5 it remained the same. But these differences failed to achieve statistical significance.

In patients treated with tamoxifen the results were similar for diploid and 'other DNA-aneuploid' tumours. When tumours with 'tetraploid' DNA peaks were considered there were changes between the two histograms. In these patients there was a striking difference between the 'tetraploid' peaks obtained from the needle biopsy and those from the resected tumours, and an example of the change is shown (Figure 2). The 'tetraploid' peak was greatly diminished in a majority of tumours, and 15 of the 20 tumours were of diploid histogram pattern in the second sample (Figure 3). These differences were highly significant (Chi-square = 20.97, $P<0.0001$).

The number of nuclei in the diploid G2+M or second major peak in each resected tumour, was expressed as a ratio to the same peak in the Trucut biopsy (Figure 4). In patients who had not received tamoxifen the median ratios for all three tumour groups were around unity, reflecting minimal change only between each pair of samples. For patients who had received tamoxifen, the median ratios for diploid and 'other DNA aneuploid' tumours were also around unity. In the tumours with a 'tetraploid' peak treated with tamoxifen there was median ratio of 5, reflecting the decrease in the 'tetraploid' peaks. Twelve of the fifteen originally 'tetraploid' tumours which became diploid in the resected tumour were positive for ER and for PR (Figure 5). Two 'tetraploid' tumours in the group which was not treated with tamoxifen also become diploid in the resected specimen. Both were positive for ER and for PR. There was a very low incidence of receptor positivity within the 'multiploid' tumours – 3 of 10 were ER positive, and 1 of 10 was PR positive.

*Diploid S phase fraction and 'tetraploid' peaks*

The diploid S phase fraction in 40 tumours with a DNA index of 2.0 was estimated by means of a computer program which is in wide use (Fried, 1976; Blackledge *et al.*, 1980). The diploid S phase fraction was related to the number of

**Figure 2** Changes in the DNA histogram after tamoxifen in a tumour with a tetraploid population of cells.

nuclei in the diploid G2+M/tetraploid G1 peak for tumours derived both from the advanced study and also from the needle biopsies of the primary operable study. The number of nuclei in the diploid S phase fractions and in the G2+M/tetraploid G1 peaks were highly correlated ($P<0.003$, unpaired $t$ test). The increase in the mean diploid S phase in shown with increase of the G2+M/tetraploid G1 peak relative to the diploid G1 peak in DNA histogram patterns (Figure 6). For this calculation the 40 histograms have been supplemented with 9 histograms from tetraploid colonic tumours.

### Discussion

Trucut biopsy samples provided adequate tissue for DNA ploidy analysis by flow cytometry: two or three 30 μm sections were taken from each sample and it was not difficult to obtain 5,000 nuclei after dissociation of the tissue. It has been estimated that a single 30 μm section of tissue 1 $cm^2$ can

**Figure 3** Changes in the number of nuclei in G2/M or second major peaks between pairs of tumours treated with tamoxifen. Each pair of tumours is represented by a single line.

**Figure 4** Ratios of number of nuclear particles in G2/M or second major peaks from resected tumours compared with needle biopsies in tumours not treated with tamoxifen.
Receptor status is shown.

**Figure 5** Ratios of number of nuclear particles in G2/M or second major peaks from resected tumours compared with needle biopsies in tumours treated with tamoxifen.
Receptor status is shown.

| Group | No. Samples | Ratio G1/G2 | S Phase Mean |
|---|---|---|---|
| A | 13 | >4 | 241 |
| B | 13 | 3–4 | 267 |
| C | 17 | 1–2 | 349 |
| D | 6 | <1 | 538 |

**Figure 6** Estimation of S phase fractions and their relationship to size of G2 peaks.

yield up to $5 \times 10^6$ nuclei (Coon *et al.*, 1986). Thus an average trucut sample ($2 \times 0.2 \times 0.1$ cm) could release $2.5 \times 10^5$ nuclei from one 30 $\mu$m section.

Tamoxifen was given as a loading dose so that therapeutic levels were present at the time of operation, and this was confirmed by measurement of serum drug concentration.

The difficulty of interpretation of a 'tetraploid' peak has been discussed in the preceding study (Baildam *et al.*, 1987). We found no consistent effect of tamoxifen on diploid and 'other DNA-aneuploid' tumours, but in 'tetraploid' tumours (near-tetraploid and tetraploid) with a significant 'tetraploid' peak ($\geqq 10\%$ nuclei), after tamoxifen in the majority there was a highly significant reduction in the number of nuclei in the 'tetraploid' peak. The possibility that the difference between the two samples might result from heterogeneity of the tumours must be considered, but this seems unlikely because no consistent changes were found in those which were untreated. In previous studies variations in DNA content were not found between primary tumours and axillary metastases (Auer *et al.*, 1984; Erhardt & Auer, 1986*a*). Variations within tumours were present in one study in two of eight tumours when multiple biopsies were taken (Prey *et al.*, 1985), and in another report, in two of seventeen tumours (Erhardt & Auer, 1986*b*). It seems unlikely that the reduction of the 'tetraploid' peaks in this study could have resulted from other than a direct or indirect effect of tamoxifen.

This study emphasises the difficulty of interpretation of 'tetraploid' DNA peaks. It is impossible to distinguish by flow cytometry between the relative contribution of diploid G2+M and tetraploid G1 nuclei to the peak. It may be postulated that many tumours with apparent 'tetraploid' peaks are essentially diploid tumours with a high proportion of cells partially arrested in the G2+M phases of the cell cycle. These 'tetraploid' cells, if generated from diploid or near-diploid cells, could represent highly dynamic and therefore potentially therapeutically-sensitive cell populations. However, it is also conceivable that in some tumours, the 'tetraploid' population consists of non-malignant cells (such as adjacent duct epithelium and/or stromal cells) with a high G2M. Inhibition of cell-cycle progression by tamoxifen in G1 (or early G2) would allow the 'tetraploid' population of cells to pass either through mitosis and enter the apparent diploid population, or to die (Taylor *et al.*, 1983*a*; Brunner *et al.*, 1985). One study demonstrated that 'tetraploid' peaks in histograms obtained from oestradiol-synchronised MCF-7 cell lines, diminished dramatically after the cultures had been exposed to tamoxifen for sixteen hours (Sutherland *et al.*, 1984). The correlation between the proportion of cells in diploid S phase and G2+M in tetraploid tumours with a DNA index of 2.0 supports this suggestion. Further experimental evidence for a diploid-tetraploid interdependence and induced diploid polyploidisation has been reported in cycling cell populations treated with cytostatic agents (Tobey *et al.*, 1978).

The reason for the postulated block in G2 is not clear. Administration of oestradiol to immune-deprived mice who bear the T61 human mammary cell line results in cessation of tumour growth and a dramatic increase in the number of cells in diploid S and G2+M, and a concurrent increase in a tetraploid S and G2+M, described as 'polyploidisation' (Brunner *et al.*, 1983). It is possible that the 'tetraploid' peaks could be produced by the effect of endogenous oestradiol causing accumulation in G2: such an accumulation could be reversed by tamoxifen. Furthermore the tetraploid G2+M peaks could be the result of 'polyploidisation' of diploid cells.

The diminution of the 'tetraploid' peak in response to tamoxifen is consistent with most of these tumours being endocrine responsive. This hypothesis is supported by the demonstration that patients with advanced disease had a high probability of response to tamoxifen if their primary tumour contained a 'tetraploid' peak (Baildam *et al.*, 1987).

However, it should be noted that tamoxifen has oestrogenic, as well as antioestrogenic effects in the first few weeks of treatment, which account for flare reactions observed clinically. Oestrogens have been found to produce cell cycle effects in breast cancer patients which are apparently independent of receptor status and perhaps mediated indirectly via an effect on host cells (cf. Conte *et al.*, 1985; Haslam, 1986).

When the diploid G2+M was <10%, there was no detectable effect of tamoxifen upon the S or G2 phases of diploid tumours. It is possible that in diploid tumours tamoxifen may inhibit proliferation by an effect in both G1 and G2. This has been suggested previously for MCF-7 cells treated with tamoxifen *in vitro*, and in the T61 tumours grown in immune-deprived mice (Brunner *et al.*, 1983; Lykkesfeldt *et al.*, 1984; Osborne *et al.*, 1983).

It cannot be determined at this stage whether clinical response to therapy corresponds to changes in the DNA histograms. Indeed the effect reported here may be completely independent of clinical response and the mechanisms whereby response is produced. We can only reiterate that in our study on advanced disease patients with 'tetraploid' tumours did demonstrate the highest response rates to endocrine manipulation, and did fare better than those with 'other DNA-aneuploid' tumours. The series of patients with primary operable disease is being closely monitored and more information will become available in time with regard to relapse and response to systemic treatment.

A.D.B. was in receipt of a grant from the Cancer Research Campaign. J.Z. is a Visiting Fellow of the Paterson Institute for Cancer Research.

### References

AUER, G., ERIKSSON, E., AZAVEDO, E., CASPERSSON, T. & WALLGREN, A. (1984). Prognostic significance of nuclear DNA content in mammary adenocarcinomas in humans. *Cancer Res.*, **44**, 394.

BAILDAM, A.D., ZALOUDIK, J., HOWELL, A. & 4 others (1987). DNA analysis by flow cytometry, response to endocrine treatment and prognosis in advanced carcinoma of the breast. *Br. J. Cancer*, **55**, this issue.

BLACKLEDGE, G., SWINDELL, R., HODGSON, B.W. & CROWTHER, D. (1980). Computerised acquisition and analysis of flow cytometric data. *Int. J. Biomed. Comput.*, **11**, 41.

BRUNNER, N., SPANG-THOMSEN, M., VINDELOV, L., WOLFFS, J. & ENGELHOLM, A. (1983). Effect of tamoxifen on the receptor-positive T61 and the receptor-negative T60 human breast carcinomas grown in nude mice. *Eur. J. Cancer. Clin. Oncol.*, **21**, 1349.

BRUNNER, N., SPANG-THOMSEN, M., VINDELOV, L., NIELSON, A., ENGELHOLM S.A. & SVENSTRUP B. (1985). Dose-dependent effect of 17$\beta$-estradiol determined by growth curves and flow cytometric DNA analysis of a human breast carcinoma (T61) grown in nude mice. *Expl. Cell Biol.*, **53**, 220.

CONTE, P.F., FRASCHINI, G., ALAMA, A. & 5 others (1985). Chemotherapy following estrogen-induced expansion of the growth fraction of human breast cancer. *Cancer Res.*, **45**, 5926.

COULSON, P.B., THORNTHWAITE, J.T., WOOLLEY, T.W., SUGARBAKER, E.V. & SECKINGER, D. (1984). Prognostic indicators including DNA histogram type, receptor content, and staging related to human breast cancer patient survival. *Cancer Res.*, **44**, 4187.

DEAN, S.W. & FOX, M. (1983). Investigation of the cell cycle response of normal and Fanconi's anaemia fibroblasts to nitrogen mustard using flow cytometry. *J. Cell Science*, **64**, 265.

ERHARDT, K. & AUER, G. (1986*a*). Mammary carcinoma: comparison of DNA content in the primary tumor and the corresponding axillary lymphnode metastases. *Acta Path. Microbiol. Immunol. Scand.* (Sect. A), **94**, 29.

ERHARDT, K. & AUER, G. (1986*b*). Mammary carcinoma: DNA analysis in areas showing different histological features in the same tumor. *Acta Path. Microbiol. Immunol. Scand.* (Sect A), **94**, 21.

FOSSA, S.D., THORUD, E., SHOAIB, M.C., PETTERSEN, E.O., HOIE, J. & SCOTT KNUDSON, O. (1984). DNA flow cytometry in primary breast carcinoma. *Acta Path. Microbiol. Immunol. Scand.* (Sect. A), **92**, 475.

FRIED, J. (1976). Method for quantitative evaluation of data from microfluorometry. *J. Comp. Biomed. Res.*, **9**, 263.

GOLANDER, Y. & STERNSON, L. (1980). Paired-ion chromatographic analysis of tamoxifen and two major metabolites in plasma. *J. Chromatog.*, **181**, 41.

HARLAND, R.N.L., HAYWARD, E. & BARNES, D.M. (1983). Progesterone receptor measurement by iso-electric focusing: a potential microassay. *Clin. Chem. Acta*, **133**, 159.

HASLAM, S.Z. (1986). Mammary fibroblast influence on normal mouse mammary epithelial cell responses to estrogen *in vitro*. *Cancer Res.*, **46**, 310.

HEDLEY, D.W., FRIEDLANDER, M.L., TAYLOR, I.W., RUGG, C.A. & MUSGROVE, E.A. (1983). Method for analysis of cellular DNA content of paraffin-embedded pathological material using flow cytometry. *J. Histochem. Cytochem.*, **31**, 1333.

HIDDEMAN, W., SCHUMANN, J., ANDREEF, M. & 6 others (1984). Convention on nomenclature for DNA cytometry. *Cytometry*, **5**, 445.

LLOYD, E.J., BARNES, D.M. & SKINNER, L.G. (1982). Isoelectric focusing of oestradiol receptor protein from human mammary carcinoma – a comparison with the dextran coated charcoal assay. *J. Steroid Biochem.*, **16**, 239.

LYKKESFELDT, A.E., LARSEN, J.K., CHRISTENSEN, I.J. & BRIAND, P. (1984). Effects of the antioestrogen tamoxifen on the cell cycle kinetics of the human breast cancer cell line, MCF-7. *Br. J. Cancer*, **49**, 717.

OSBORNE, C.K., BOLDT, D.H. & ESTRADA, P. (1984). Human breast cancer cell cycle synchronization by estrogens and antiestrogens in culture. *Cancer Res.*, **44**, 1433.

PREY, M.U., MEYER, J.S., STONE, K.R. & McDIVITT, R.W. (1985). Heterogeneity of breast carcinomas determined by flow cytometric analysis. *J. Surg. Oncol.*, **29**, 35.

ROCHEFORT, H., BORGNA, J.L., COEZY, E., VIGNON, F. & WESTLEY, B. (1981). Mechanism of action of tamoxifen and metabolites in MCF-7 human breast cancer cells. In *Non-steroidal anti-oestrogens*, Sutherland, R.L. & Jordan, V.C. (eds) p. 355. Academic Press: Sydney.

STUART-HARRIS, R., HEDLEY, D.W., TAYLOR, I.W., LEVENE, A.L. & SMITH, I.E. (1985). Tumour ploidy, response and survival in patients receiving endocrine therapy for advanced breast cancer. *Br. J. Cancer*, **51**, 573.

SUTHERLAND, R.L., GREEN, M.D., HALL, R.E., REDDEL, R.R. & TAYLOR, I.W. (1983). Tamoxifen induces accumulation of MCF-7 human mammary carcinoma cells in the $G_0/G_1$ phase of the cell cycle. *Eur. J. Cancer Clin. Oncol.*, **19**, 615.

SUTHERLAND, R.L., MURPHY, L.C., HALL, R.E., REDDEL, R.R., WATTS, C.K.W. & TAYLOR, I.W. (1984). Effects of antioestrogens on human breast cancer cells *in vitro*. Interactions with high affinity intracellular binding sites and effects on cell proliferation kinetics. *Prog. Cancer Res. Ther.*, **31**, 193.

TAYLOR, I.W. & MILTHORPE, B.K. (1980). An evaluation of DNA fluorochromes, staining techniques and analysis for flow cytometry. *J. Histochem. Cytochem.*, **28**, 1224.

TAYLOR, I.W., HODSON, P.J., GREEN, M.D. & SUTHERLAND, R.L. (1983). Effects of tamoxifen on cell cycle progression of synchronous MCF-7 human mammary carcinoma cells. *Cancer Res.*, **43**, 4007.

TOBEY, R.A., DEAVEN, L.L. & OKA, M.S. (1978). Kinetic response of cultured Chinese hamster cells to treatment with 4′-[(9-Acridinyl)-amino]methanesulphon-*m*-anisidide-HCl. *J. Natl Cancer Inst.*, **60**, 1147.